# Abundance, arrangement, and function of sequence motifs in the chicken promoters

**DOI:** 10.1186/1471-2164-15-900

**Published:** 2014-10-15

**Authors:** Hideaki Abe, Neil J Gemmell

**Affiliations:** Department of Anatomy, University of Otago, Dunedin, New Zealand; Allan Wilson Centre for Molecular Ecology and Evolution, University of Otago, Dunedin, New Zealand

**Keywords:** Promoter, Transcription factor binding site, Short tandem repeat, CpG island, G-quadruplex, Gene ontology, Transcription, GC content, Selection, *Cis*-regulatory element

## Abstract

**Background:**

Eukaryotic promoters are regions containing various sequence motifs necessary to control gene transcription. Much evidence has emerged showing that structural and/or contextual changes in regulatory elements can critically affect *cis*-regulatory activity. As sequence motifs can be key factors in maintaining complex promoter architectures, one effective approach to further understand the evolution of promoter regions in vertebrates is to compare the abundance and distribution patterns of sequence motifs in these regions between divergent species. When compared with mammals, the chicken (*Gallus gallus*) has a very different genome composition and sufficient genomic information to make it a good model for the exploration of promoter structure and evolution.

**Results:**

More than 10% of chicken genes contained short tandem repeat (STR) in the region 2 kb upstream of promoters, but the total number of STRs observed in chicken is approximately half of that detected in human promoters. In terms of the STR motif frequencies, chicken promoter regions were more similar to other avian and mammalian promoters than these were to the entire chicken genome. Unlike other STRs, nearly half of the trinucleotide repeats found in promoters partly or entirely overlapped with CpG islands, indicating potential association with nucleosome positions. Moreover, the chicken promoters are abundant with sequence motifs such as poly-A, poly-G and G-quadruplexes, especially in the core region, that are otherwise rare in the genome. Most of sequence motifs showed strong functional enrichment for particular gene ontology (GO) categories, indicating roles in regulation of transcription and gene expression, as well as immune response and cognition.

**Conclusions:**

Chicken promoter regions share some, but not all, of the structural features observed in mammalian promoters. The findings presented here provide empirical evidence suggesting that the frequencies and locations of STR motifs have been conserved through promoter evolution in a lineage-specific manner. Correlation analysis between GO categories and sequence motifs suggests motif-specific constraints acting on gene function.

**Electronic supplementary material:**

The online version of this article (doi:10.1186/1471-2164-15-900) contains supplementary material, which is available to authorized users.

## Background

Promoters are well-characterized transcriptional *cis*-regulatory sequences in complex genomes [1]. They generally locate immediately upstream of a transcription start site (TSS) and have a variety of sequence motifs that participate in gene regulation [2]. The key elements include transcription factor binding sites (TFBSs), short tandem repeat (STR), G-quadruplex (G4), and CpG island (CGI), which are frequently co-localized and otherwise integrated into combined motifs. Given these *cis*-regulatory DNA sequences contain TFBSs and/or other regulatory modules that play a critical role in transcription, mutations that either alter affinity of TFBSs or disrupt spacing between existing TFBSs have the potential to affect *cis*-regulatory activity [3, 4]. Throughout the last decade, empirical data have accumulated suggesting that mutations in regulatory elements could be a major cause of phenotypic divergence [5–7].

Recent studies have shown that eukaryotic promoters are rich in repetitive sequences — approximately 25% of yeast (*Saccharomyces cerevisiae*) genes contain at least one STR in their regulatory elements [8]. In humans, the Short Tandem Repeats in Regulatory Regions Table (STaRRRT) has shown that 5,264 STRs are present in the upstream regulatory region of 4,441 genes [9]. Recently, genes driven by repeat-containing promoters are reported to have significantly higher rates of transcriptional divergence than those without repeat elements, as corroborated by *in vitro* experiments showing that the gain or loss of STR units within promoters yields quantitative differences in gene expression [8, 10, 11]. For example, the EWS/FLI protein, which belongs to EST-type transcription factor (TF), directly binds to GGAA-motifs in the glutathione S-transferase M4 promoter, and transcriptional activity is highly dependent upon the number of repeats [12–14]. These facts, together with a higher conservation rate of STRs in regions proximal to TSSs than that in distal regions [15], strongly support a significant role for tandem repeats in differential transcriptional regulation.

Other DNA sequence motifs that affect chromatin structure have the potential to impact on gene expression by changing the accessibility of transcription and regulatory proteins to the DNA. G4 is one such sequence motif that has a four-stranded DNA structure held together by four or more tandem guanine tracts [16]. Recent work has shown that ~60% of the genes in warm-blooded animals represented by human, mouse, and chicken have at least one potential G-quadruplex sequence (PQS) within the 5 kb region upstream of TSS [17]. Some of the G4 sequences so far examined appear to act as silencer elements in the promoter regions [18]. The clearest evidence for a role of G4 structure in transcriptional regulation comes from empirical study of the onco-gene *c-myc* [19]. Disruption of G4 motifs in the *c-myc* promoter resulted in increased gene expression, whereas stabilization of the G4 decreased transcription, raising the strong possibility that G4 formation affects the deposition of regulatory proteins and histones on double-stranded DNA [17, 18].

CpG islands are CG-rich stretches that have been found in approximately half of mammalian promoters at or near the TSS [20]. In vertebrates, promoters with CGI are characterized by the presence of many TSS and high transcriptional activity in multiple tissues, whereas promoters without CGIs are defined by a single TSS and show tightly regulated expression in specific tissues [21, 22]. Correlations between gene ontologies (GOs) and CGI length hint at the important role of CGI in higher-order chromatin structures via methylation [23]. Most of the CGIs in chicken promoters remain hypomethylated, contributing to nucleosome-free regions over the promoter [24]. Open CGI/CG-rich promoters would naturally lack nucleosome scaffolds that are required to adopt an open conformation, and different histone modification patterns have been observed between genes with or without CGI promoters [25].

Stochastic and spatial data on the aforementioned sequence motifs that may modify chromatin structure and affect transcription are essential for understanding the nature of regulatory complexity in higher organisms. In this study we investigate the enrichment and arrangement of several sequence motifs within chicken (*Gallus gallus*) promoters to shed light on compositional structure in avian promoter sequences and their association with gene functions. Birds are hypothesized to have diverged from a common ancestor with mammals around 300 million years ago [26]. Thus, it is of interest to investigate whether previous findings on the distribution and abundance of sequence motifs in mammalian promoters would be consistent with those derived from chicken promoters, given the significant divergence times and very different evolutionary trajectories these lineages have followed.

## Results

### GC content and CpG island density

**Figure 1:**
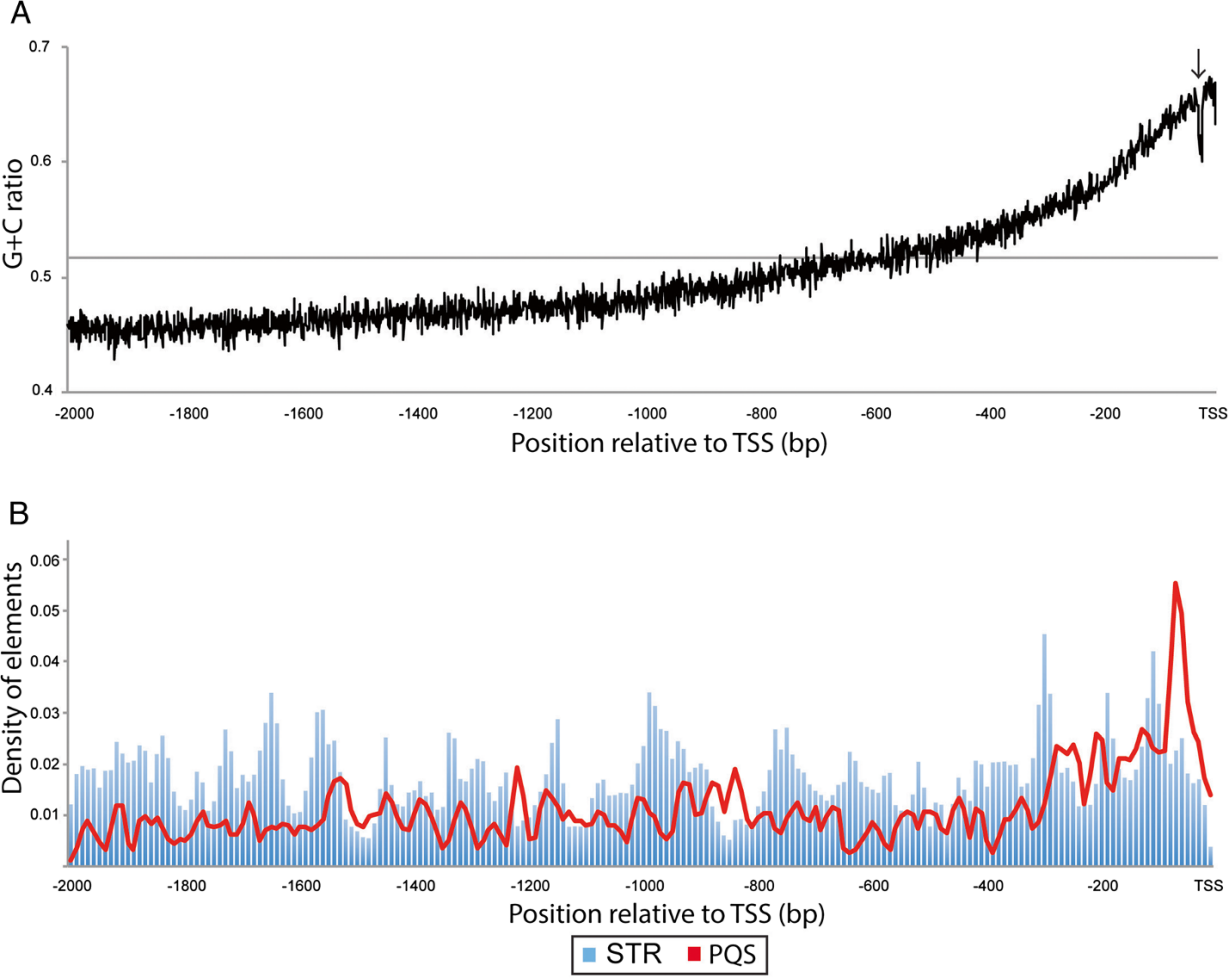
**GC contents and the distribution of sequence motifs in the chicken promoter. (A)** The GC contents in the upstream regulatory regions. Horizontal line and arrow indicate the average GC ratio and expected position of TATA-box, respectively. **(B)** The number of each sequence motif was counted in 10 bp bins as shown in blue bars (Short tandem repeat; STR) and red line (Potential G-quadruplex sequence; PQS).

Chicken promoter sequences had considerable variation in their GC contents, ranging from 31.9 to 73.6%, and their overall average was 51.5% (Additional file 1). The average G + C ratio increased gradually as it approached the TSS (Figure 1A). A sharp decline detected in the core promoter region (-31 to -23 bp) was presumably attributed to the presence of a TATA box. The Newcpgreport software identified 2,809 CGIs in promoter regions, and the number of CGIs-containing genes reached to 2,251 (58.3%). This was almost the same with that reported elsewhere [27], even though search conditions were not exactly the same. The distribution of CpG observed/expected (O/E) ratio was slightly lower (mode O/E = 0.95 ~ 1.00; Additional file 2) as compared with the previous report [22] (mode O/E = 1.1 ~ 1.2). This might be due to the fact that some of CGIs identified in this study were truncated at TSS. We referred to the chicken promoters harboring long CGIs (>800 bp in total) as “long CGI” (LCGI) promoters, because they occupied the top10% of the genes in terms of CGI length. “No CGI” (NCGI) promoter was hereafter used to refer to promoters without CGI.

### Abundance of short tandem repeats and G-quadruplexes in chicken promoters

Table 1 summarizes the frequencies of STR motifs in chicken and other mammalian promoters as well as those identified in the entire chicken genome. The total number of STRs observed in chicken promoters was almost equivalent to that observed in both duck and zebra finch promoters. In all avian species, approximately 10% of genes had at least 6 perfect repeat units of 2–6 nucleotide STR in 2 kb upstream region from TSS, whereas mouse and human promoters contained a much larger number of STRs in the same regions. The number of STR motifs counted per human promoter sequence was more than double that of chicken promoter. A rank correlation analysis showed that the STR motif frequency in chicken promoter shares a significant level of similarity with all the promoter sets examined here (Table 1). However, the comparison of STR motif frequencies between chicken promoter and the entire chicken genome exhibited the lowest similarity values in all statistical parameters (i.e., correlation coefficient, Kendall’s *τ* distance, and 2-sided *p*-value for Kendall *τ* rank correlation), confirming the paucity of STRs previously described for this genome [28]. Several STR motifs also showed taxon-specific differences in their distribution. For example, avian promoters were distinguishable from those of mammals on the basis of a lower frequency of AG/CT motifs. The duck promoter contained an extremely low number of GC-rich STR motifs such as CG and CCG.

**Table 1:** **Comparison of STR motif distributions among chicken and avian/mammalian promoters**

	Promoter										Genome*	
	Chicken		Duck		Zebra finch		Mouse		Human		Chicken	
Assembly	***galGal4***		***BGI_duck_1.0***		***taeGut3.2.4***		***mm10***		***hg19***		***galGal3***	
Motif	Count	Freq.	Count	Freq.	Count	Freq.	Count	Freq.	Count	Freq.	Count	Freq.
AC	405	(25.9)	433	(31.5)	378	(23.2)	6006	(40.0)	3189	(35.6)	97994	(26.1)
AT	276	(17.6)	294	(21.4)	270	(16.6)	739	(4.9)	887	(9.9)	52845	(14.1)
AG	174	(11.1)	174	(12.7)	186	(11.4)	2572	(17.1)	1356	(15.1)	98988	(26.4)
AGG	131	(8.4)	98	(7.1)	170	(10.4)	426	(2.8)	192	(2.1)	13509	(3.6)
AAT	68	(4.3)	61	(4.4)	40	(2.5)	264	(1.8)	413	(4.6)	18974	(5.1)
CCG	66	(4.2)	1	(0.1)	87	(5.3)	200	(1.3)	462	(5.2)	722	(0.2)
AAC	46	(2.9)	38	(2.8)	16	(1.0)	356	(2.4)	283	(3.2)	13218	(3.5)
AGC	40	(2.6)	26	(1.9)	72	(4.4)	73	(0.5)	67	(0.7)	31737	(8.5)
AAAT	30	(1.9)	49	(3.6)	21	(1.3)	432	(2.9)	393	(4.4)	6017	(1.6)
AAAG	26	(1.7)	21	(1.5)	10	(0.6)	583	(3.9)	223	(2.5)	2769	(0.7)
CG	26	(1.7)	0	(-)	36	(2.2)	177	(1.2)	207	(2.3)	687	(0.2)
ATCCC	18	(1.2)	6	(0.4)	14	(0.9)	1	(0.0)	0	(-)	ND	
AAAC	17	(1.1)	33	(2.4)	14	(0.9)	563	(3.7)	183	(2.0)	6290	(1.7)
AAGG	15	(1.0)	11	(0.8)	13	(0.8)	274	(1.8)	122	(1.4)	1094	(0.3)
AAG	13	(0.8)	9	(0.7)	5	(0.3)	246	(1.6)	75	(0.8)	15000	(4.0)
ACC	13	(0.8)	8	(0.6)	8	(0.5)	150	(1.0)	224	(2.5)	8154	(2.2)
AGAGG	13	(0.8)	5	(0.4)	7	(0.4)	54	(0.4)	0	(-)	ND	
ATCC	12	(0.8)	5	(0.4)	32	(2.0)	41	(0.3)	29	(0.3)	436	(0.1)
ACGGC	12	(0.8)	0	(-)	12	(0.7)	0	(-)	0	(-)	ND	
AAGGG	11	(0.7)	22	(1.6)	6	(0.4)	23	(0.2)	4	(0.0)	ND	
STR/seq**	0.114		0.101		0.104		0.555		0.231		–	
vs.chicken promoter												
Correlation	*r*	–	0.985		0.984		0.852		0.912		0.841	
Kendall	*τ*	–	0.595		0.738		0.581		0.656		0.487	
	*p*	–	< 0.001		< 0.0001		< 0.001		< 0.0001		< 0.05	

*Data derived from [50]. ND: no data available.

**The number of STR motif counted per promoter sequence.

Chicken STRs were not equally distributed, but rather varied over the region of promoters (Figure 1B). A total of 302 PQSs were identified in the chicken promoters but unlike STRs, PQSs were especially accumulated in the core promoter region. The number of PQS identified in this study was much fewer than that previously reported in transcriptional regulatory region of chicken genome [29]. This was probably due to differences in the stringency of PQS screening as well as in length of target promoter region.

### Heterogeneity in the pattern of STR expansion between avian and mammalian promoters

**Figure 2:**
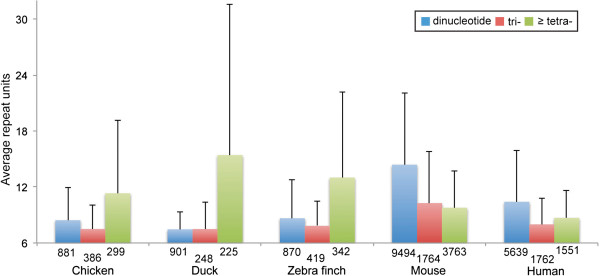
**The average number of STR units detected in avian and mammalian promoters.** Note that only perfect repeats with at least six units are considered here. The number of STR loci is described under each bar. The total number of promoter sequences for five organisms are: chicken (13,754), duck (13,583), zebra finch (15,633), mouse (27,046), and human (38,687).

The pattern of STR unit expansion was quite different between avian and mammalian promoters. All avian promoters examined here exhibited a similar trend of STR expansion with significantly larger number of STR units in tetra-, penta-, and hexanucleotide (hereafter tetranucleotide) repeats against dinucleotide repeats (Figure 2; Mann–Whitney U-test [chicken]; z =5.56, *p* <0.001, [duck]; z =7.33, *p* <0.001, [zebra finch]; z =10.75, *p* <0.001). While human and mouse promoters were characterized with much longer dinucleotide repeats as compared with the tetranucleotide repeats (Mann–Whitney U-test [human]; z =6.14, *p* <0.001, [mouse]; z =27.72, *p* <0.001). The number of dinucleotide repeat units was significantly lower in chicken promoters than that of human (Mann–Whitney U-test; z =8.69, *p* <0.001), but the reverse was true for tetranucleotide repeats, which are prevalent in chicken versus human (Mann–Whitney U-test; z =3.69, *p* <0.001).

### Distribution of sequence motifs in conjunction with CpG islands

**Figure 3:**
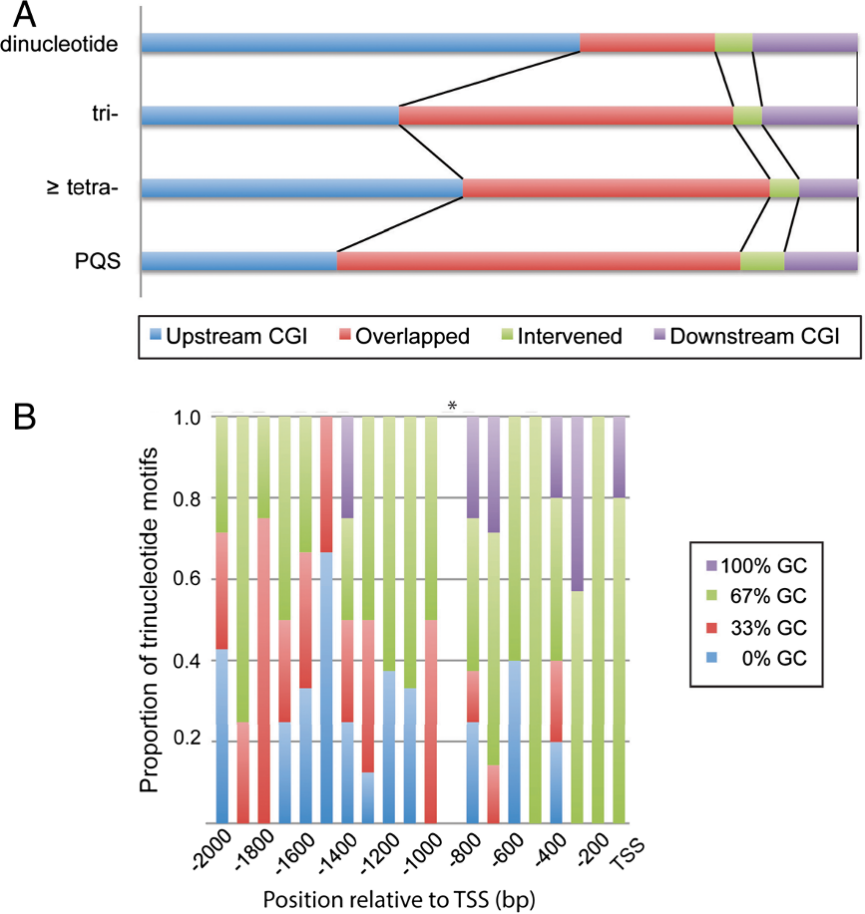
**Biases in the location of sequence motifs found in the chicken promoter. (A)** The locations of STRs and PQSs are shown in conjunction with a CpG island (CGI). Upstream and downstream CGI indicate the locations of sequence motifs. “Overlapped” means that at least part of a sequence motif is overlapped with a CGI. “Intervened” is used when a sequence motif is found to be flanked by two or more CGIs (not overlapped). There is significant difference in the locations between di- and trinucleotide repeats (Fisher’s exact test; *p* <0.001). **(B)** The distribution of trinucleotide repeats in chicken promoter. Trinucleotide repeats are classified into four categories by the number of guanine or cytosine in each repeat unit. Note that no trinucleotide repeat was found in the position at -901 to -1000 bp (asterisk).

Some chicken promoters contained multiple sequence motifs, which either co-existed with or were integrated with CGIs. The maximum number of CGI, STR, and PQS identified in a single promoter was 5, 5, and 4, respectively. The relative abundance of STRs did not change the rate of multiple CGIs, and *vice versa*. However, the co-existence of PQSs significantly affected the CGI number in promoter regions (Fisher’s exact test (FET); *p* <0.001; Additional file 3). The number of STRs that overlapped with CGI motifs was significantly different between di- and trinucleotide repeats (FET; *p* <0.001; Figure 3A). Most of dinucleotide STRs were located upstream of CGIs, whereas a higher proportion of trinucleotide repeats was found to be overlapped with CGIs. We anticipated that almost all of PQSs would be found to overlap with CGIs, but actually 43.7% of PQSs located in up- or down-stream of the CGI.

### Low repeat number and positional bias of trinucleotide repeats in chicken promoter

The average number of trinucleotide repeat units were the smallest both in avian and mammalian promoters in all STR periods. All chicken trinucleotide repeats were divided into four groups that had different numbers of guanine or cytosine bases in each repeat unit (hereafter referred as 100%GC, 67%GC, 33%GC, and 0%GC). Figure 3B shows different pattern of trinucleotide STR distribution between 100%GC and low GC group (33% and 0%GC; FET; *p* <0.01). The motifs with 100%GC distributed mostly in the proximal and core regions, while 33% and 0%GC motifs were predominantly found in the distal part of promoter.

### Conserved motifs identified in the chicken promoter

**Figure 4:**
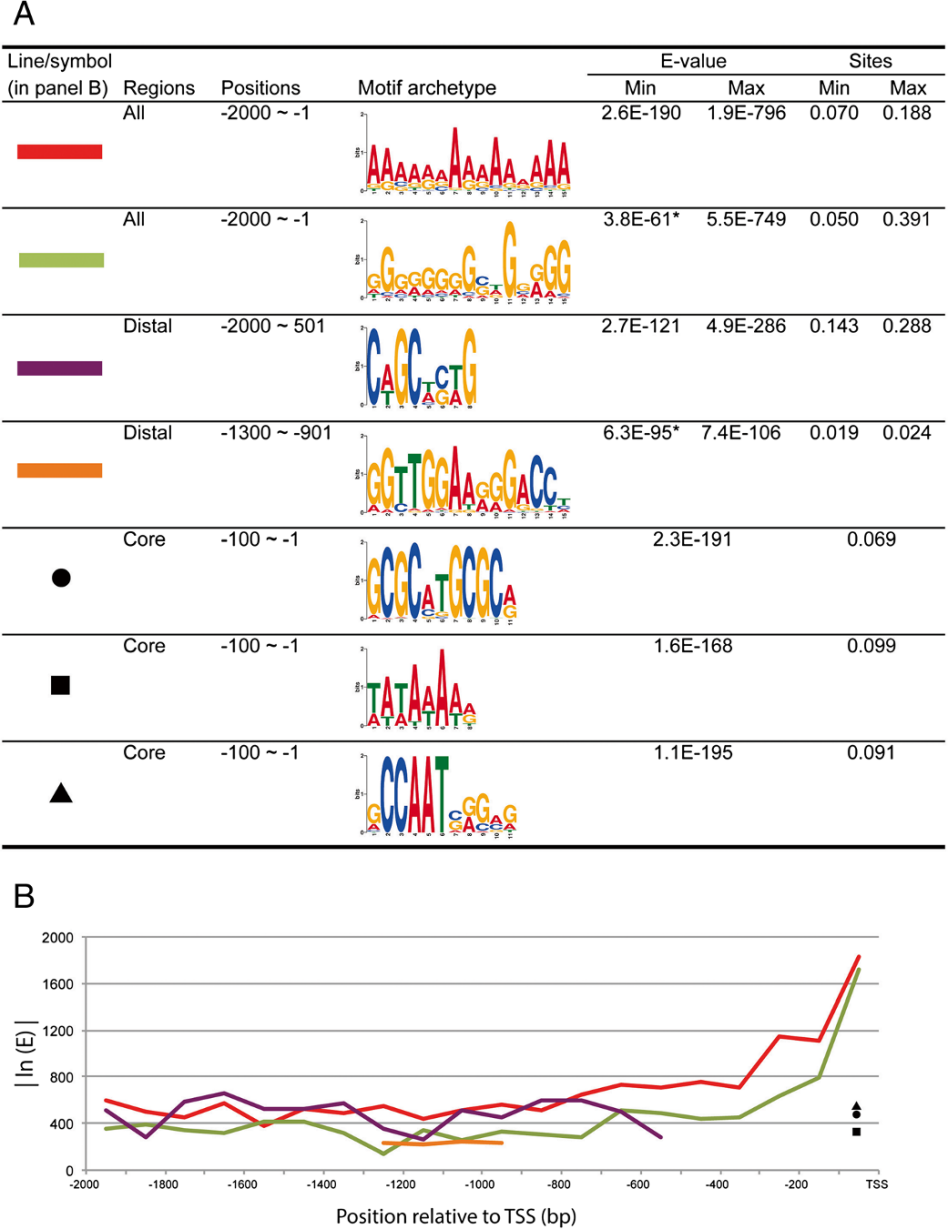
**Conserved motifs identified by MEME Suite software.** Chicken promoter sequences were divided into 100 bp bins and used for MEME search. **(A)** Each archetype of motifs is shown with the maximum and minimum E-values and the proportion of motif identified in a dataset. Cut-off E-value was set in 1.0E-100 except two cases (shown with asterisk). **(B)** The distribution of conserved motifs in the upstream regulatory regions.

The MEME Suite [30] was used to detect conserved motifs that might affect gene regulation. The most and second most common blocks in the chicken promoter were poly-A and poly-G prevalent in promoter regions (Figure 4A and B). Both polypurine repeats were relatively constant in the motif frequencies through the distal promoter (-2000 to -500 bp), but gradually increased through the proximal region (-500 to -100 bp). Both motifs were characterized by a steep increase in the core promoter region (-100 bp to TSS). The other motif C[A/T]GC[A/T][C/G][A/T]G also appeared in the distal promoter, but was seldom seen either in proximal or in core regions. This motif was compared to known motifs in JASPAR Vertebrates [31] and UniPROBE Mouse database [32] by TOMTOM [33] ver 4.9.1. As a result, the following 10 TF motifs were detected with significant level of similarities (FET; *p* <0.01); Zfp691, TFAP4, Zic1, NHLH1, Zbtb3, Zic3, ZEB1, Osr2, Tcf3, and Gfi1b. Approximately 10% of chicken promoters contained a TATA box in their core region and the number and location of TATA boxes in the chicken promoters were comparable to those reported in the genome-wide analysis of mammalian promoters, showing -30 and -31 from TSS as the preferred sites [34].

### Gene functions associated with sequence motifs

**Figure 5:**
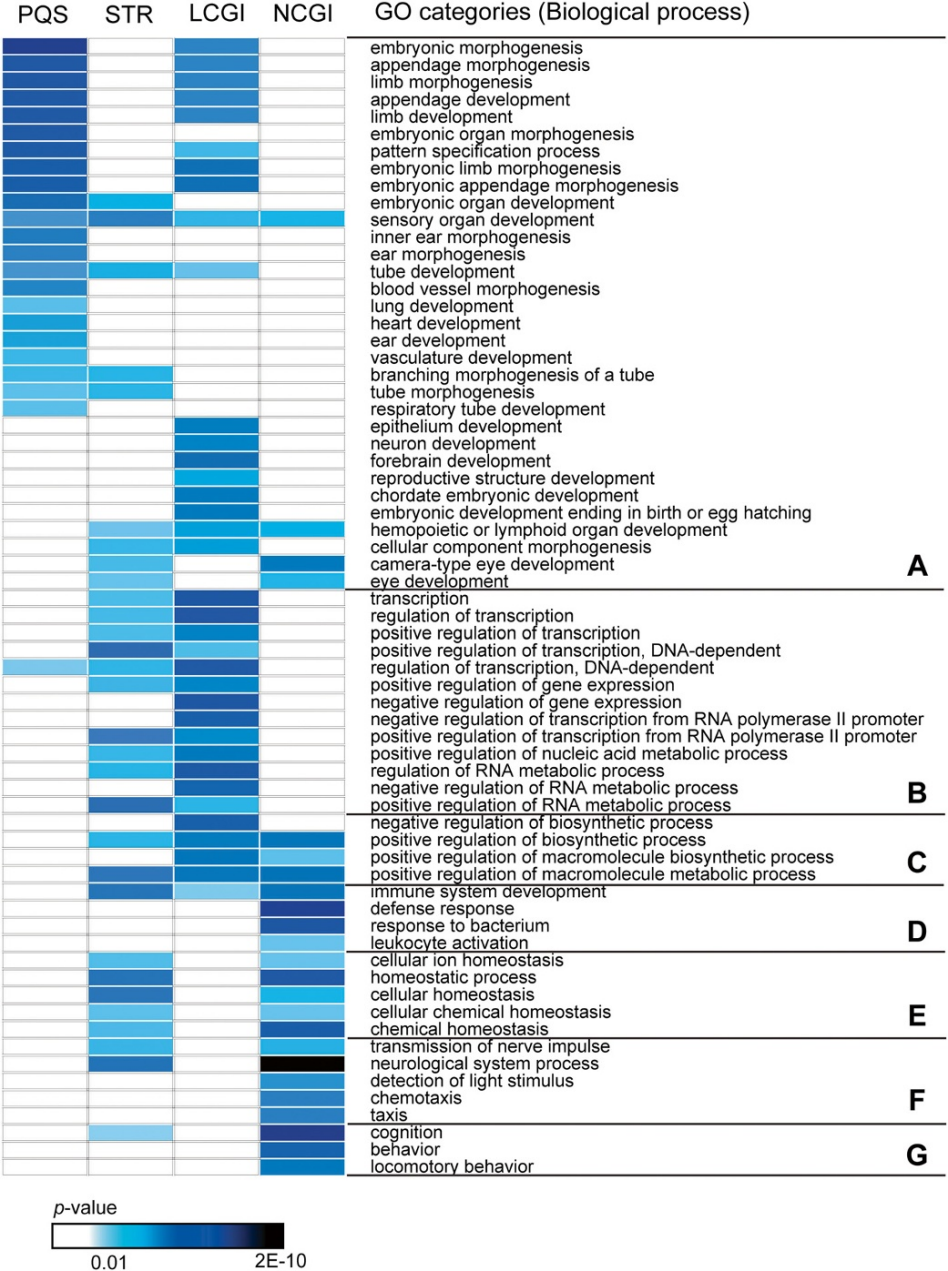
**Correlation between gene ontology (GO) and sequence motifs identified in the chicken promoter.** Statistical significance of the correlation between GO and each sequence motif is represented in a heat map with cut-off *p*-value of 0.01. LCGI and NCGI indicate long CpG island (>800 bp) and no CpG island, respectively. Biological processes are hierarchically grouped into seven upper categories: **(A)** development and morphogenesis; **(B)** regulation of transcription/RNA metabolic process; **(C)** regulation of biosynthetic process; **(D)** immune response; **(E)** homeostasis; **(F)** response to stimulus; **(G)** cognition and behavior.

For functional annotation analysis using the Database for Annotation, Visualization, and Integrated Discovery (DAVID) program [35], chicken promoters were grouped into four sets of genes depending on the presence or absence of sequence motifs (i.e., PQS, STR, LCGI, and NCGI). The heat map shown in Figure 5 clearly illustrates a bias in biological processes that exhibit significant probabilities. PQSs were predominantly detected in the genes associated with development and morphogenesis, while genes with STRs were less correlated with the particular GO terms. LCGI promoters were strongly associated with gene functions related to regulation of transcription and gene expression, whereas NCGI promoters associated with other gene functions such as immune response and cognition. A full list of GO categories found to be correlated with the sequence motifs is presented in Additional file 4.

## Discussion

In the present study, we show that chicken promoter sequences share some, but not all features with the human and mouse promoters. Although the frequency and variety of STR motif were highly conserved even between avian and mammalian promoters, chicken promoters had the least similarity with the entire chicken genome in terms of STR motif frequency. This finding is partly supported by previous data that showed the predominant STR motifs found in promoter region (TSS to -500 bp) were quite distinct from those detected in the other part of genes (i.e., 5′ untranslated region (UTR), coding, intron, and 3′ UTR) both in human and mouse [36]. The AC/GT was the most common motif in all the promoter sequences examined in this study, but AG/CT motifs were predominantly found in human 5′ UTR and coding regions [36]. The inconsistency of predominant STR motifs between promoter and adjacent non-promoter regions seems to support the previous suggestion that STR motifs in promoters can alter gene expression as they expand or contract, with particular attention to secondary structures [37]. Considering that (AC/GT)_n_ dinucleotide repeats have a propensity to form Z-DNA and occasionally block the movement of RNA polymerase when it occurs downstream of the TSS [38], repeat expansion or contraction of this motif might be constrained by the conformation of other sequence motifs that participate in transcriptional regulation. Hence, the AC/GT motif may be regarded as the most frequently used “tuning knob”, as demonstrated by Bayele *et al.* [39], and this role appears to be evolutionarily conserved in vertebrates.

However, there are some points of difference in the STR motif frequencies and their expansion between avian and mammalian promoters. In mammalian promoters, the number of tetranucleotide STR units was significantly lower than those of avian promoters. One possible explanation of this pattern is that the expansion of tetranucleotide repeats might have been subjected to purifying selection to preserve some functions in human promoters. Such a scenario is also consistent with the frequently reported associations between tetranucleotide expansion and human diseases [40]. For instance, alteration in array length of TAAA affected the level of *nadA* transcription through modulation of the binding of the transcription factor IHF [41]. Another study on human prostate cancer also suggested an involvement of TAAA tandem repeats as mediators of the expression of *PCA3* gene [42]. Further investigation is needed to elucidate any lineage-specific preference for STR expansion in vertebrate promoters.

Another important finding is that the density of STRs in the chicken promoter region is much lower than that estimated for human promoter. Taking into account that avian genomes contain much less STRs than mammals [43, 44], it may simply reflect the difference in the occurrence rate of slippage-like indels across organisms, as suggested by Kruglyak *et al.* [45]. In this case the higher GC contents observed in chicken promoters [46] as well as relatively small genome size [47] are plausible reasons for the lower occurrence rate of slippage events.

Several previous works have shown *cis*-regulatory motifs to be constrained in a position- and/or distance-specific manner [48, 49]. STRs are among the most plausible factors contributing to the changing spaces between functional elements in promoters. Our data clearly showed that the distribution and expansion of STR in chicken promoters are largely different among repeat unit classes. Dinucleotide tandem repeats were mainly found in non-CpG sites, whereas a higher rate of trinucleotide repeats were overlapped with CGI, with fewer repeats. Therefore, it is tempting to speculate that the expansion of trinucleotide STRs in chicken promoter is constrained either by a position and distance limitation or by direct targeting of TF. Furthermore, trinucleotide repeats showed skewed distribution between high and low ratio of guanine/cytosine in the repeat unit (Figure 3B). This finding is of great interest since the previous study on STR abundance in the chicken genome demonstrated that the rate of STR polymorphisms increases in high GC group (67% and 100%GC), exclusively in trinucleotide tandem repeats [50]. This discrepancy may be explained by the significant role of the trinucleotide tandem repeats as an enhancer/modulator of transcription in the core promoter region [51]. A previous *in vitro* experiment also supports the significance of 100% GC trinucleotide repeats as a key modulator of transcription, indicating that the insertion of (CGG)_12_ into the *CYC1- lacZ* promoter increased gene expression about 10-fold, even other trinucleotide repeats of (CTG)_12_ and (GAA)_12_ had little effect [52]. All these facts imply that trinucleotide motifs with high guanine or cytosine contents, especially those found in the proximal and core promoter regions may have a pivotal role in the maintenance of an open chromatin structure, which will constrain STR expansion. Indeed, several studies clearly illustrated that GC-rich trinucleotide repeats are highly flexibility and possess a greater propensity to bend towards the major groove [53, 54].

Motif identification using the MEME software revealed several conserved motifs either in all or in particular part of chicken promoters. The poly-A was the most ubiquitous motif among them. Previous studies indicated that polypurine motifs are the most common STRs in the human genome and are particularly enriched in promoter regions [55]. It was suggested that poly-A might act to alter the stability or dynamics of nucleosomes, somehow enhancing the ability of gene activator proteins to bind nearby DNA target sites [56]. This hypothesis is well supported by our observation that poly-A are especially abundant in the core promoter region where maintenance of open chromatin structure is necessary. In contrast, the abundance of poly-G in core promoter is likely to provide potential binding sites for Sp1, which is a crucial TF for the expression of some genes. For example, the human *vascular endothelial growth factor* (*VEGF*) promoter contains a 39-bp poly-G sequence, located -85 to -50 bp relative to the TSS, including three potential Sp1 binding sites [57]. These independent studies give strong indications that sequence motifs - TFBS associations within the core promoter region may hold the key to deciphering the complexity of gene expression. In chicken promoters, we also detected another conserved motif, C[A/T]GC[A/T][G/C][A/T]G in the distal promoter. Cooper *et al.* reported that negative elements to human promoter activity were identified -1000 to -500 bp upstream of the TSS by their deletion analyses [58]. Therefore, it is possible that the conserved motifs that are unique to the distal part of the promoter may have some role as negative regulators of promoter activity.

CGIs are deeply involved in gene regulatory processes [59]. In particular, the length of CGIs is a pivotal factor in determining the number of RNA polymerase II binding sites in mammalian promoters [21]. In this study, LCGI promoters were strongly associated with the biological processes such as “regulation of transcription” (GO: 0045449, FET; *p* <10^-7^) and “transcription” (GO: 0006350, FET; *p* <10^-7^), whereas NCGI promoters were significantly involved in gene function that linked with “neurological system process” (GO: 0050877; FET; *p* <10^-9^) and “defence response” (GO: 0006952: FET; *p* <10^-7^). In addition, some of GO categories related with development and morphogenesis were moderately associated with LCGI promoters. These findings are analogous to the results obtained in mammalian promoters [21, 22], indicating that an association between CGI lengths and particular gene functions is conserved, at least within warm-blooded vertebrates. In other words, we find that both the pattern of tissue-specific gene expression [60] and the motif-specific expression patterns are evolutionary conserved across the warm-blooded vertebrates. Another intriguing finding is that chicken promoters with PQS motifs are generally correlated with GO categories related to development and morphogenesis. It is notable that some specific biological processes such as (inner) ear morphogenesis and heart development were not significantly correlated with LCGI but with PQS mediated promoters. This observation suggests that some PQSs that are non-overlapping with CGI might play a decisive role in gene expression through the fine-tuning of transcriptional activity. This, together with the recent finding that cell proliferation/cell-cycle could be regulated by presence of PQS - TFBS combinations in mammalian promoters [61] hint at the importance of positional context of sequence motifs. The case-by-case approach should be employed to reveal the underlying role of PQSs as transcriptional regulators in chicken promoters.

## Conclusions

This paper has provided novel findings from the investigation of sequence motifs in chicken promoter. The STR motif frequency in chicken promoters is similar with both those of other avian and mammalian promoters, but relatively divergent from that of the rest of the chicken genome. We have also revealed that the pattern of STR unit expansion is largely different between avian and mammalian promoters. These findings indicate that STR sequence motifs in promoter regions are strongly conserved and may play roles in transcription regulation, but that lineage specific pressures on motif expansion may exist. Although GC content in chicken promoter is higher than in mammals, the same pattern of correlations between biological processes and CGI lengths can be found in this study. Moreover, we have shown that PQSs are exclusively recognized in a set of genes involved in development and morphogenesis. Searching for lineage-specific patterns of various sequence motifs in promoter regions will certainly extend our understanding of the relationship between structural complexity of promoters and functional consequences.

## Methods

### Data preparation

In order to compare the STR motif distributions between chicken and other animals (duck, zebra finch, mouse, and human), the 2 kb upstream sequences from flanking genes were obtained either from Ensemble BioMart [62] or from the University of California, Santa Cruz (UCSC) Genome Browser [63, 64]. In this study, we expediently defined the “promoter region” as 2 kb upstream of TSS, while acknowledging that *cis*-regulatory elements can also be found much further upstream, 3′ of the gene, or inside of genes [65]. Chicken sequences 2 kb upstream of the TSS of RefSeq genes with annotated 5′ UTRs were also downloaded from the UCSC browser and used for structural and function-related analyses. Consequently, we obtained 3,858 promoter sequences from the corresponding genes with functional annotations (up to 22.6% of total genes in the chicken genome (UCSC release *galGal4*)).

### Identification of sequence motifs

CGIs were identified using the Newcpgreport downloaded from the European Bioinformatics Institute (EMBL-EBI) Browser [66]. The traditional criteria were used to identify CGIs: (i) base composition of guanine and cytosine in a window (100 bp) exceeded 50%, (ii) minimum length was 200 bp, and (iii) the ratio of observed to expected number of CpG dinicleotides (CpG O/E) was more than 0.6 [67]. We did not try to seek 3′ end of CGIs when they were overlapping with TSS and reached to 5′ UTRs. Thus, some of CGIs identified in this study were truncated up to several hundreds bp in length.

The avian and mammalian promoter sequences were screened for STRs using the WebSat [68] and Phobos software [69] integrated into the STADEN package [70]. We searched STRs under following conditions: (i) only perfect repeats were considered, (ii) repeats periods were 2, 3, 4, 5, and 6, (iii) STRs with at least six repeat units were scored, and (iv) combined STRs with two or more motifs were counted separately. We did not take mononucleotide repeats into consideration, mainly due to their uncertainty in the repeat number. The data on STR frequency distributions were subjected to the non-parametric Kendall *τ* trend analysis between chicken and other animals under the null hypothesis of no association between two data sets. The bottom 10% of minor motifs in chicken STR frequency were eliminated from the data set. In addition, information on STR occurrence in the entire chicken genome was obtained from the previous study [50] and compared with those detected within promoter regions to examine the heterogeneity of STR motif frequency between them.

PQSs were detected by the Quadruplex forming G-Rich Sequences (QGRS) Mapper software [71]. The details of search parameters were as follows: (i) max length of PQSs was 30, (ii) the minimum number of tetrads in a G4 was four, and (iii) the minimum loop size was set to zero. Note that these parameters led some motifs being double-counted in both STRs and PQSs. For example, the (GGGGT)_6_ motif found in the *distal-less homeobox 3* (*DLX3*) gene was hit by both STRs and PQSs searches. The motifs that comprised of undisrupted poly-G were not counted as PQS motifs.

### Motifs discovery by the MEME Suite

The MEME Suite was used to find sequence motifs representing features such as DNA binding sites and protein interaction domains on the promoter regions [30]. The promoter sequences were divided into 100 bp bin to create query files. MEME has a large number of optional inputs to fine-tune its performance. The following options were used: (i) zero or one occurrence per sequence model (i.e., zoops) was chosen, (ii) the maximum width of the motifs was 15, (iii) motifs occurrences were on the given DNA strand or on its reverse complement (i.e., revcomp), and (iv) the number of motifs was set to five. The probability reported by MEME is actually an approximation of the E-value of the log likelihood ratio, and the width of the motifs can also affect the statistical significance of the motifs. Thus, in general, motifs with a longer width tend to have lower levels of E-value in MEME analysis.

### Gene ontology analysis

The analysis of functional gene annotations was performed using the DAVID ver. 6.7 available on website [35, 72]. We sorted chicken promoter sequences into four subgroups based on presence or absence of the aforementioned target motifs. The sequence motifs that shared characteristics with both PQSs and STRs were sorted into the STR. FET *p*-values were calculated to estimate the level of over-representation of the selected genes in GO categories [73], especially in the biological process. Probabilities less than 0.01 were used as cut-off value and considered to show significant level of correlation. Heat map analysis was also conducted through DAVID outcomes to visualize a matrix of enriched GO. R software ver. 3.0.2 was used to create heat map of the significances.

## Electronic supplementary material

Additional file 1: **GC content of each chicken promoter sequence.** The GC content of each promoter shown with the overall average GC content (horizontal bar). (PDF 958 KB)

Additional file 2: **The observed/expected (O/E) CpG ratio in the chicken promoter.** The maximum O/E CpG ratio plotted against the number of genes. (PDF 388 KB)

Additional file 3: **Co-occurrence of CpG island and sequence motifs in a single promoter.** The number of chicken genes was statistically examined whether it showed significant excess of co-occurring motif pairs. (PDF 61 KB)

Additional file 4: **A full list of genes found to be correlated with sequence motifs.** GO biological process categories enriched amongst genes, either of which has a sequence motif within a promoter. (PDF 33 KB)

## Abbreviations

bp: Base pairs
CGI: CpG island
DAVID: Database for annotation, visualization, and integrated discovery
EBI: European bioinformatics institute
FET: Fisher’s exact test
G4: G-quadruplex
GO: Gene ontology
LCGI: Long CpG island
NCGI: No CpG island
O/E: Observed/expected
PQS: Potential G-quadruplex sequence
QGRS: Quadruplex forming G-rich sequences
STaRRRT: Short tandem repeats in regulatory region table
STR: Short tandem repeat
TF: Transcription factor
TFBS: Transcription factor binding site
TSS: Transcription start site
UCSC: University of California, Santa Cruz
UTR: Untranslated region.
