# Supplementary figures and images for: Abundance, arrangement, and function of sequence motifs in the chicken promoters
Source: BMC Genomics. 2014 Oct 15;15(1):900. doi: 10.1186/1471-2164-15-900 (PMC4203960; doi:10.1186/1471-2164-15-900)

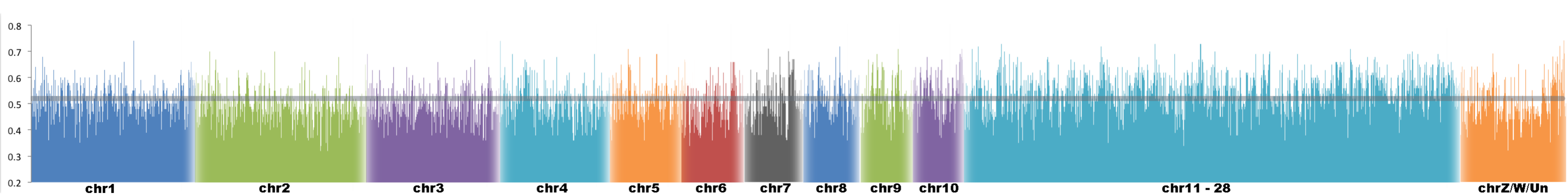

Additional file 1

Supplement: Supplementary file 1 — Additional file 1: GC content of each chicken promoter sequence. The GC content of each promoter shown with the overall average GC content (horizontal bar). (PDF 958 KB) [file 12864_2014_6586_MOESM1_ESM.pdf]

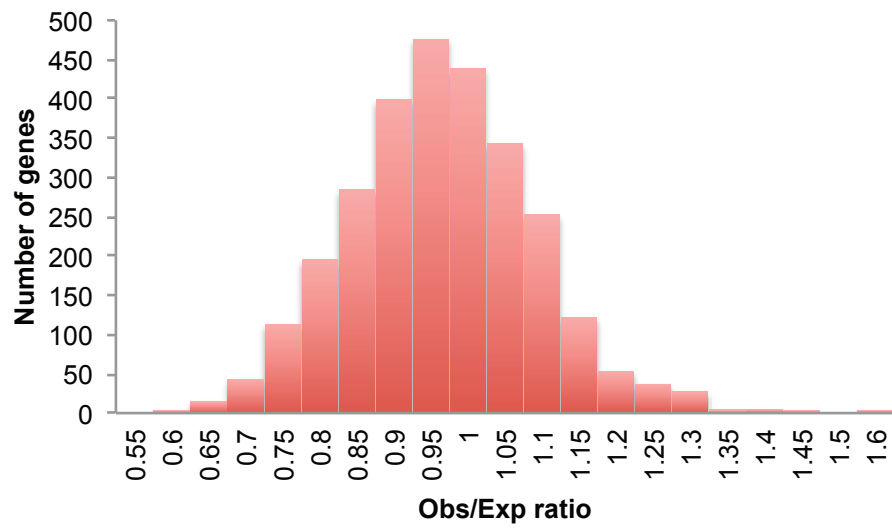

Additional file 2

Supplement: Supplementary file 2 — Additional file 2: The observed/expected (O/E) CpG ratio in the chicken promoter. The maximum O/E CpG ratio plotted against the number of genes. (PDF 388 KB) [file 12864_2014_6586_MOESM2_ESM.pdf]
