# Supplementary material for: Abundance, arrangement, and function of sequence motifs in the chicken promoters
Source: BMC Genomics. 2014 Oct 15;15(1):900. doi: 10.1186/1471-2164-15-900 (PMC4203960; doi:10.1186/1471-2164-15-900)
Supplement: Supplementary file 3 — Additional file 3: Co-occurrence of CpG island and sequence motifs in a single promoter. The number of chicken genes was statistically examined whether it showed significant excess of co-occurring motif pairs. (PDF 61 KB) [file 12864_2014_6586_MOESM3_ESM.pdf]

Additional file 3

Co-occurrence of CpG island and sequence motifs in a single promoter (shaded)

(Number of genes)

	#	CGI 0	CGI 1	CGI 2	CGI 3	CGI 4	CGI 5	*p*-value
STR	0	1433	1599	336	55	9	1	
	1	143	151	37	9	3	0	n.s.
	2	25	24	8	4	1	0	n.s.
	3	4	8	0	1	0	0	n.s.
	4	3	2	0	1	0	0	—
	5	0	1	0	0	0	0	—
p-value			n.s.	n.s.	0.001	—	—	n.s.*
PQS	0	1553	1665	316	56	10	1	
	1	54	107	53	10	3	0	2.2E-14
	2	1	10	6	3	0	0	1.8E-05
	3	0	1	3	1	0	0	—
	4	0	2	3	0	0	0	—
p-value			2.0E-05	5.3E-20	6.1E-09	—	—	1.2E-20*

* Overall probability is calculated under the null hypothesis that the number of CGI has no correlation with the existence of each sequence motif (significant *p*-value is less than 0.01).
