# Supplementary material for: Abundance, arrangement, and function of sequence motifs in the chicken promoters
Source: BMC Genomics. 2014 Oct 15;15(1):900. doi: 10.1186/1471-2164-15-900 (PMC4203960; doi:10.1186/1471-2164-15-900)
Supplement: Supplementary file 4 — Additional file 4: A full list of genes found to be correlated with sequence motifs. GO biological process categories enriched amongst genes, either of which has a sequence motif within a promoter. (PDF 33 KB) [file 12864_2014_6586_MOESM4_ESM.pdf]

Additional file 4

GO	Biological process	PQS	MS_all	CGI_800	CGI_none
GO:0042692	muscle cell differentiation	0.002356385			0.013522936
GO:0008283	cell proliferation	0.006061144			6.36E-06
GO:0006928	cell motion	0.031817764			0.015279271
GO:0001775	cell activation				3.81E-04
GO:0044093	positive regulation of molecular function	0.046893257	0.020139422		
GO:0042127	regulation of cell proliferation	1.50E-04	2.75E-05	2.51E-04	4.99E-04
GO:0050678	regulation of epithelial cell proliferation	0.038893475		0.028298926	
GO:0050679	positive regulation of epithelial cell proliferation			0.027462813	0.021843525
GO:0040008	regulation of growth	0.043251634		0.045777662	0.018932777
GO:0051270	regulation of cell motion	0.006815005		0.048058454	0.008247982
GO:0008284	positive regulation of cell proliferation	0.010625707	2.18E-04	0.00151115	0.012470532
GO:0008285	negative regulation of cell proliferation	0.00691972	0.015004839	0.007736812	
GO:0042981	regulation of apoptosis		0.016053613	4.52E-04	0.008029074
GO:0010941	regulation of cell death		0.009049716	6.14E-04	0.012236911
GO:0012501	programmed cell death		0.049189199	0.030300652	0.04855894
GO:0016265	death		0.013778896		0.015462095
GO:0042981	regulation of apoptosis		0.016053613	4.52E-04	0.008029074
GO:0010941	regulation of cell death		0.009049716	6.14E-04	0.012236911
GO:0012501	programmed cell death		0.049189199	0.030300652	0.04855894
GO:0008219	cell death		0.012275974	0.047705052	0.012366157
GO:0060548	negative regulation of cell death			0.014110424	0.037739159
GO:0043067	regulation of programmed cell death		0.008658259	5.78E-04	0.011274749
GO:0048666	neuron development	0.041409263	0.029482205	5.21E-04	0.016812257
GO:0030182	neuron differentiation	0.00880551	0.01282013	0.001127073	0.014610835
GO:0019226	transmission of nerve impulse		0.002089803		0.004852425
GO:0050877	neurological system process		1.96E-04		2.55E-10
GO:0042110	T cell activation	0.032620919			0.026717898
GO:0045321	leukocyte activation	0.049371521		0.035148798	0.00427584
GO:0050863	regulation of T cell activation		0.022618879		0.015608008
GO:0050867	positive regulation of cell activation		0.022618879		0.015608008
GO:0050870	positive regulation of T cell activation		0.049930178		0.012079164
GO:0002694	regulation of leukocyte activation		0.010233022		0.042695117
GO:0051249	regulation of lymphocyte activation		0.029587488		0.026717898
GO:0002696	positive regulation of leukocyte activation		0.018624169		0.026691666
GO:0051172	negative regulation of nitrogen compound metabolic process			5.30E-05	
GO:0051173	positive regulation of nitrogen compound metabolic process		0.002417051	3.81E-04	0.005620799
GO:0031327	negative regulation of cellular biosynthetic process		0.030359853	3.39E-06	
GO:0031328	positive regulation of cellular biosynthetic process		0.001630762	2.67E-04	9.99E-04
GO:0031399	regulation of protein modification process	0.040377582			0.023575684
GO:0032268	regulation of cellular protein metabolic process	0.038770592	0.015415936		0.019146382
GO:0010720	positive regulation of cell development	0.01178811			
GO:0010740	positive regulation of protein kinase cascade				4.54E-04
GO:0009967	positive regulation of signal transduction		0.049547224		7.20E-07
GO:0009968	negative regulation of signal transduction		0.016304053		0.018959632
GO:0010647	positive regulation of cell communication				2.23E-06
GO:0010648	negative regulation of cell communication		0.020947541		0.030014537
GO:0044093	positive regulation of molecular function	0.046893257	0.020139422		
GO:0050678	regulation of epithelial cell proliferation	0.038893475		0.028298926	
GO:0050679	positive regulation of epithelial cell proliferation			0.027462813	0.021843525
GO:0051174	regulation of phosphorus metabolic process	0.007232803			0.00128557
GO:0019220	regulation of phosphate metabolic process	0.007232803			0.00128557
GO:0042325	regulation of phosphorylation	0.005580773			0.001313091
GO:0007548	sex differentiation			0.024894391	0.022230918
GO:0019953	sexual reproduction		0.013071656		1.66E-04
GO:0042698	ovulation cycle		0.030933014	0.005872114	
GO:0045137	development of primary sexual characteristics		0.02480911	0.03311462	
GO:0048609	reproductive process in a multicellular organism		0.00988689	0.015762767	0.001618696
GO:0022602	ovulation cycle process		0.030933014	0.005872114	
GO:0048511	rhythmic process		0.034353834	0.001882023	
GO:0046148	pigment biosynthetic process		0.018790057		0.015554861
GO:0019748	secondary metabolic process				0.029328361
GO:0016055	Wnt receptor signaling pathway		0.028429299	0.039246571	
GO:0010033	response to organic substance			0.00398056	0.001646904
GO:0016044	membrane organization		0.006554519	0.030859949	0.030529574
GO:0016192	vesicle-mediated transport			0.015194753	0.002065641
GO:0032504	multicellular organism reproduction		0.00988689	0.015762767	0.001618696
GO:0007010	cytoskeleton organization				9.79E-04
GO:0051046	regulation of secretion				2.40E-04
GO:0051051	negative regulation of transport		0.001064888	0.03945115	0.030934989
GO:0042330	taxis	0.046361725			6.64E-04
GO:0030097	hemopoiesis		0.008614094	0.004632191	0.002625673
GO:0040012	regulation of locomotion	0.001193127		0.04205948	0.01350932
